# Coenzyme Q10 Ameliorates Pancreatic Fibrosis via the ROS-Triggered mTOR Signaling Pathway

**DOI:** 10.1155/2019/8039694

**Published:** 2019-02-07

**Authors:** Ran Xue, Jianxin Wang, Lixin Yang, Xinjuan Liu, Yan Gao, Yanhua Pang, Yanbin Wang, Jianyu Hao

**Affiliations:** Department of Gastroenterology, Beijing Chao-Yang Hospital, Capital Medical University, Beijing 100020, China

## Abstract

**Aim:**

Pancreatic stellate cells (PSCs) play a pivotal role in pancreatic fibrosis. Any remedies that inhibit the activation of PSCs can be potential candidates for therapeutic strategies in pancreatic fibrosis-related pancreatic ductal adenocarcinoma (PDAC) and chronic pancreatitis (CP). Our study is aimed at exploring the protective effect of coenzyme Q10 (CoQ10) against pancreatic fibrosis.

**Methods:**

Pancreatic fibrosis was induced by 20% L-arginine (250 mg/100 g) at 1 h intervals twice per week for 8 weeks in C57BL/6 mice. CoQ10 was administered for 4 weeks. Isolated primary PSCs from C57BL/6 mice were treated with 100 *μ*M CoQ10 for 72 h, as well as Rosup and specific inhibitors. The effects of CoQ10 on the activation of PSCs, autophagy, collagen deposition, histological changes, and oxidative stress were analyzed by western blotting, biochemical estimations, immunofluorescence staining, and hematoxylin-eosin, Masson, and Sirius red staining, as well as with a reactive oxygen species (ROS) assay.

**Results:**

Pretreatment and posttreatment of CoQ10 decreased autophagy, activation of PSCs, oxidative stress, histological changes, and collagen deposition in the CP mouse model. In primary PSCs, expression levels of p-PI3K, p-AKT, and p-mTOR were upregulated with CoQ10. A rescue experiment using specific inhibitors of the PI3K-AKT-mTOR pathway demonstrated that the PI3K-AKT-mTOR signaling pathway was the underlying mechanism by which CoQ10 ameliorated fibrosis. With the addition of Rosup, expression levels of the autophagy biomarkers LC3 and Atg5 were elevated. Meanwhile, the levels of p-PI3K, p-AKT, and p-mTOR were lower.

**Conclusions:**

Our findings demonstrated that CoQ10 alleviates pancreatic fibrosis by the ROS-triggered PI3K/AKT/mTOR signaling pathway. CoQ10 may be a therapeutic candidate for antifibrotic methods.

## 1. Introduction

In recent years, several lines of evidence have suggested that pancreatic stellate cells (PSCs) play pivotal roles in the development of pancreatic fibrosis, which is conceivably a dynamic process involved in the transition to pancreatic ductal adenocarcinoma (PDAC) [1]. In a normal pancreas, PSCs are quiescently localized in the periacinar region, while displaying retinoid-containing droplets and compounding low amounts of extracellular matrix (ECM) proteins. When PSCs are activated, they transform into a myofibroblast-like phenotype that can be recognized by the existence of alpha-smooth muscle actin (*α*-SMA), various kinds of growth factors and cytokines, and a large amount of ECM proteins [2, 3]. Furthermore, the colocalization of major ECM proteins and *α*-SMA appeared to be associated with the degree of fibrogenesis [4, 5]. In short, the activation of PSCs is vital to the development of pancreas-related diseases, including PDAC and chronic pancreatitis (CP).

Therefore, in-depth study of the processes related to PSC activation is important for the development of efficient therapeutic methods for pancreas-related diseases. Any remedies that can suppress the activation of PSCs may be potential candidates for clinical therapy strategies in PDAC and CP.

Many studies have shown that oxidative stress occurs in PDAC and CP, proven through increased levels of products of oxidative stress and reduced maintenance of antioxidant capacity in patients with PDAC and CP [6, 7]. Furthermore, antioxidant supplementation can lower oxidative stress levels in patients with alcoholic and idiopathic CP [8, 9]. Animal studies demonstrated a cessation of the fibrotic cascade with antioxidant supplement treatment. Antioxidant therapy also reduced high glucose-induced PSC activation in PSCs [10].

Coenzyme Q10 (CoQ10) is a naturally occurring coenzyme with powerful antioxidant effects that is involved in electron transport in the mitochondria, as well as being an anti-inflammatory agent [11, 12]. Our recent study found that in primary mouse PSCs, CoQ10 suppressed the activation of PSCs [13]. It appeared that the PI3K/AKT/mTOR signaling pathway participated in this process. To provide more groundwork regarding CoQ10 in the treatment of PSC-related diseases, we further studied the effect of CoQ10 on pancreatic fibrosis in a C57BL/6 mouse model, as well as the related molecular mechanisms in this study.

## 2. Materials and Methods

### 2.1. Animals

Healthy male C57BL/6 mice (15-30 g) from Beijing Chao-Yang Hospital, Capital Medical University, were used. They were acclimated for 2 weeks before experimentation. The investigations were conducted according to ethical standards and the Declaration of Helsinki. This study was also approved by the Beijing Chao-Yang Hospital, Capital Medical University. All methods and procedures including animals were performed according to the guidelines of the Animals Committee.

### 2.2. Experimental Design, Animal Treatment, and Dose Selection

Animals were randomized into 6 groups. Each group included 10 animals. CP was induced by 20% L-arginine hydrochloride (250 mg/100 g) in normal saline in C57BL/6 mice (18–30 g) through i.p. injection at 1 h intervals twice per week for 8 weeks. Normal controls received normal saline at 1 h intervals twice per week for 8 weeks. CP animals received 20% L-arginine hydrochloride (250 mg/100 g) in normal saline at 1 h intervals twice per week for 8 weeks. CoQ10 pretreatment animals received 20% L-arginine hydrochloride (250 mg/100 g) in normal saline at 1 h intervals twice per week for 8 weeks. After 4 weeks, animals received CoQ10 in the drinking water until the end of the 8^th^ week. CoQ10 posttreatment animals received 20% L-arginine hydrochloride (250 mg/100 g) in normal saline at 1 h intervals twice per week for 8 weeks, with CoQ10 in the drinking water for 5 weeks. Both L-arginine (L-Arg) and CoQ10 were dissolved in saline freshly at the time of injection.

### 2.3. Reactive Oxygen Species (ROS) Staining

Pancreas tissues (5 mm^3^ in volume) were cut into pieces to obtain single cells using single-cell suspension preparation equipment. Nylon membrane (200-mesh) was used to filter the single-cell suspension. The suspension was centrifuged at 4000 *g* for 10 min at 4°C. The single-cell precipitates were resuspended, and the supernatants were discarded. The ROS fluorescent probe 2′,7′-dichlorofluorescein diacetate (DCFH-DA, Sigma, USA) was added to the precipitates and incubated for 30 min in a 37°C incubator. Stained single-cell suspensions were centrifuged at 12,000 *g* for 8 min. The precipitates were washed with phosphate buffered saline (PBS). The fluorescence intensities of the single-cell suspensions were measured at random using flow cytometry.

### 2.4. Hematoxylin-Eosin (H&E), Masson, and Sirius Red Staining

Routine H&E, Masson, and Sirius red staining were performed as described in a previous study [14]. The results were assessed independently and blindly by two investigators.

### 2.5. Measurement of GSH, SOD, and MDA Content

Serum GSH (U/L), SOD (U/mL), and MDA (nmol/mL) levels were measured according to methods previously described [15]. GSH, SOD, and MDA levels were calculated by a standard reference curve using reduced glutathione as a standard.

### 2.6. Immunofluorescence (IF) Staining

Tissues were incubated with primary antibodies (Supplementary Materials (available here)). Immunofluorescence was photographed using a confocal laser scanning microscope (Leica, Heidelberg, Germany).

### 2.7. Isolation of PSCs and CoQ10 Treatment

C57BL/6 PSCs were isolated and cultured as described [16]. In each experiment, PSCs were seeded at 1 × 10^5^ cells/mL, and CoQ10 was added at 100 *μ*M (FBS) with the conditions of incubation temperature 37°C and 5% CO_2_ for 72 h. Then, cells were collected for analysis.

### 2.8. Western Blotting Analysis

Western blotting analysis was performed as described [17]. Samples of 16–50 *μ*g of total protein were used for western blotting. The list of primary antibodies is displayed in Supplementary Materials. Protein bands were visualized using West Pico Chemiluminescent Substrate (Thermo Fisher Scientific, USA).

### 2.9. Statistical Analyses

Statistical significance was calculated using Student's *t*-test (SPSS19.0, Chicago, USA). Quantitative variables are expressed as the mean ± S.D. for at least three experiments. *P* < 0.05 was considered significant.

## 3. Results

### 3.1. The Effect on the Pancreas Weight, Body Weight, and Morphological Characteristics

**Figure 1:**
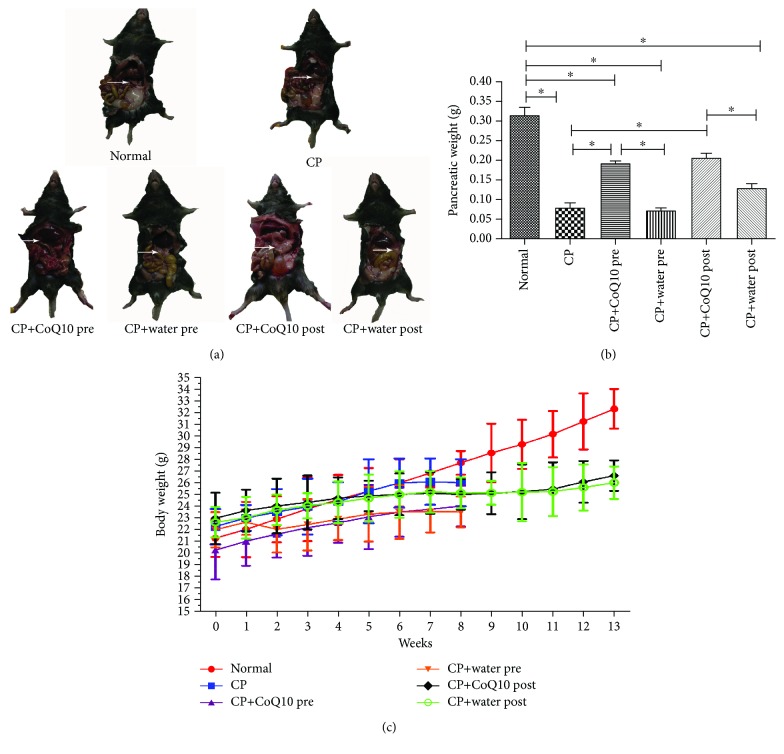
(a) Representative images of the morphology of the pancreas in various treatment groups. (^∗^The white arrow is placed next to the pancreas.) (b) Effect of CoQ10 and CP on the absolute pancreas weight (^∗^ *P* < 0.05; *N* = 3). (c) Effect of CoQ10 and CP on the body weight with the time changing.

In the CP group, the pancreas appeared abnormal in morphology, showing adhesion to surrounding tissues, with decreased and reduced pancreatic tissue mass (Figure 1(a)). Compared to the CP group, pretreatment and posttreatment with CoQ10 showed a smoother surface of the pancreas, softer in texture and less adherent to the surrounding tissue, as well as increased pancreatic tissue weight (Figure 1(b)). In the CP group, the growth rate of mice over time slowed down and weight loss even occurred. However, pretreatment and posttreatment with CoQ10 restored body weight compared to the respective controls (*P* < 0.05) (Figure 1(c)).

### 3.2. The Effect on Oxidative Stress

**Figure 2:**
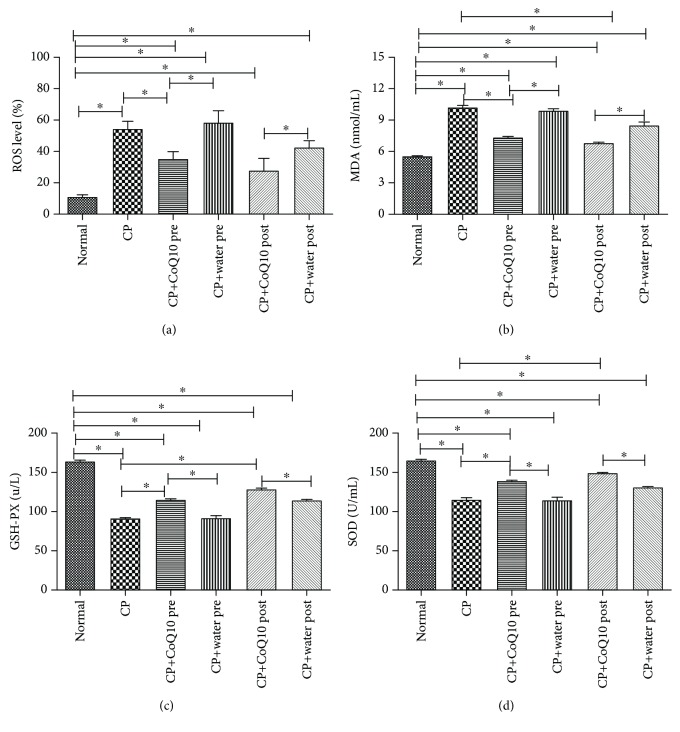
(a) ROS levels were tested using flow cytometry by the DCFH-DA fluorescent probe. There were significant differences between the CoQ10-treated group and the CP group in terms of ROS levels (^∗^ *P* < 0.05; *N* = 3). (b) The MDA levels were reduced with CoQ10 treatment compared with the CP group (^∗^ *P* < 0.05; *N* = 3). (c) The GSH-PX levels were increased with CoQ10 treatment compared with the CP group (^∗^ *P* < 0.05; *N* = 3). (d) The SOD levels were increased with CoQ10 treatment compared with the CP group (^∗^ *P* < 0.05; *N* = 3).

The CP group showed higher tissue ROS production compared with the normal group (*P* < 0.05). Compared with the respective CP control group, a significant decrease in tissue ROS production was observed with pretreatment and posttreatment with CoQ10 (*P* < 0.05) (Figure 2(a)). In the CP group, there were significantly higher MDA levels, whereas pretreatment and posttreatment with CoQ10 decreased those levels compared with the respective controls (Figure 2(b)). Moreover, compared with the respective controls, pretreatment and posttreatment with CoQ10 increased the L-Arg-induced decrease in GSH and SOD levels (*P* < 0.05) (Figures 2(c) and 2(d)).

### 3.3. The Effect on Histological Changes

**Figure 3:**
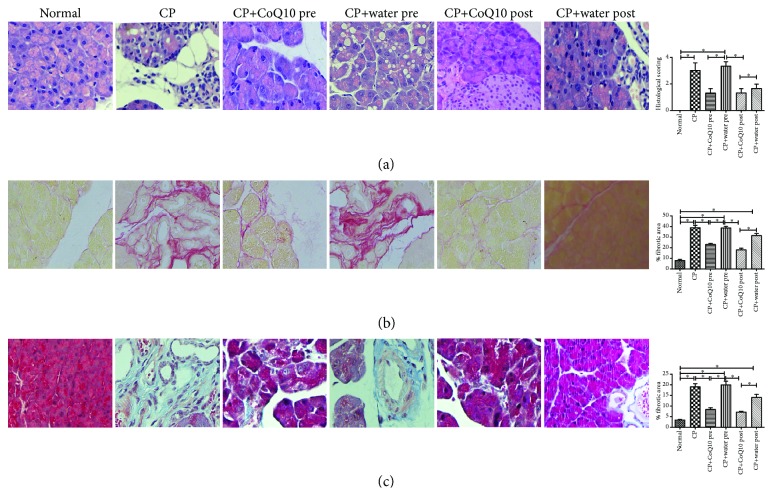
(a) Representative photomicrographs showing the effect of CoQ10 and CP histological alterations in the pancreatic tissue, stained with H&E. Histogram showing the quantitative histological evaluation of histological damage in the pancreas of different treatment groups. All values are expressed as the mean ± SEM (*n* = 3); ^∗^ *P* < 0.05. (b) Representative photomicrographs showing the effect of CoQ10 and CP on the collagen deposition in the pancreas of the various treatment groups, evaluated by Sirius red staining. Histograms showing the quantitative analysis of the percentage of fibrotic area. All values are expressed as the mean ± SEM (*n* = 3); ^∗^ *P* < 0.05. (c) Representative photomicrographs showing the effect of CoQ10 and CP on collagen deposition in the pancreas of various treatment groups, evaluated by Masson trichrome staining. Histograms showing quantitative analysis of the percentage of fibrotic area. All values are expressed as the mean ± SEM (*n* = 3); ^∗^ *P* < 0.05.

Light microscopic investigations showed that control animals had normal histological architecture of the pancreas. However, there were severe histological changes in the CP group, including inflammatory cell infiltration and vacuolization with complete loss of the cytoplasm as well as acinar cell atrophy. The pancreas in the CP group also presented with relatively more significant histological changes selectively in acinar cells. Pretreatment and posttreatment with CoQ10 resulted in a remarkable alteration of the abovementioned histological changes (Figure 3(a)).

### 3.4. The Effect on Collagen Deposition

Sirius red staining displayed more red-stained areas, validating the fibrosis and collagen deposition in the pancreas of the CP group, while pretreatment and posttreatment with CoQ10 significantly decreased the percentage of the Sirius red-positive area (red) (Figure 3(b)). Pretreatment and posttreatment with CoQ10 significantly decreased pancreatic fibrosis as demonstrated by a decrease in the percentage of fibrotic area (blue) on Masson trichrome staining compared to the respective controls (Figure 3(c)).

### 3.5. The Effect on the Activation of PSCs

**Figure 4:**
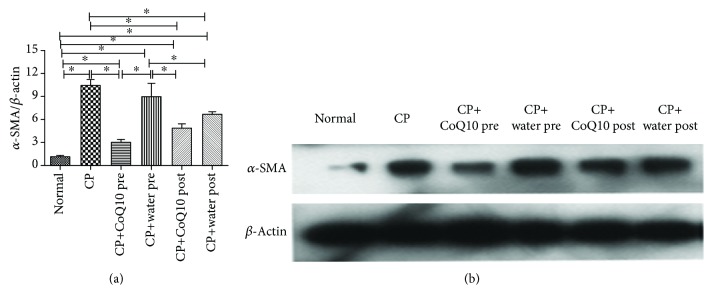
(a) Quantification of western blots for *α*-SMA expression levels in various groups compared to the normal control group (^∗^ *P* < 0.05; *N* = 3). (b) Western blotting analysis for *α*-SMA expression levels of activated PSCs was performed with CoQ10 treatment.

In western blotting analysis, compared with the normal control group, there was a significant increase in the expression of *α*-SMA. CoQ10 treatment reduced the *α*-SMA expression compared to the respective CP controls (Figure 4). Taken together, these results suggested that CoQ10 treatment inhibited the activation of PSCs.

### 3.6. The Effect on Autophagy

**Figure 5:**
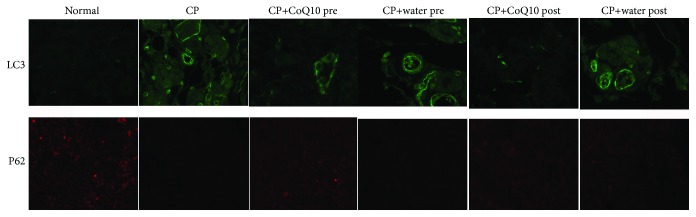
Pancreas tissue stained positively for LC3 by IF (Alexa Fluor 488 staining for LC3, original magnification, ×100). Pancreas tissue stained positively for P62 by IF (Alexa Fluor 594 staining for P62, original magnification, ×100).

As shown in Figure 5, compared with the normal control group, the CP group had a higher autophagy level in the pancreas tissue, with an increase in the levels of LC3-II and a decrease in the levels of p62. Furthermore, IF showed that pretreatment and posttreatment with CoQ10 reduced the accumulation of LC3-II and impaired p62 clearance compared with the CP group. CoQ10 induced a decrease in the levels of LC3-II and an increase in the levels of p62, indicating that CoQ10 suppressed tissue autophagy levels in CP.

### 3.7. The Underlying Mechanism of CoQ10 Suppression of the Activation of PSCs

**Figure 6:**
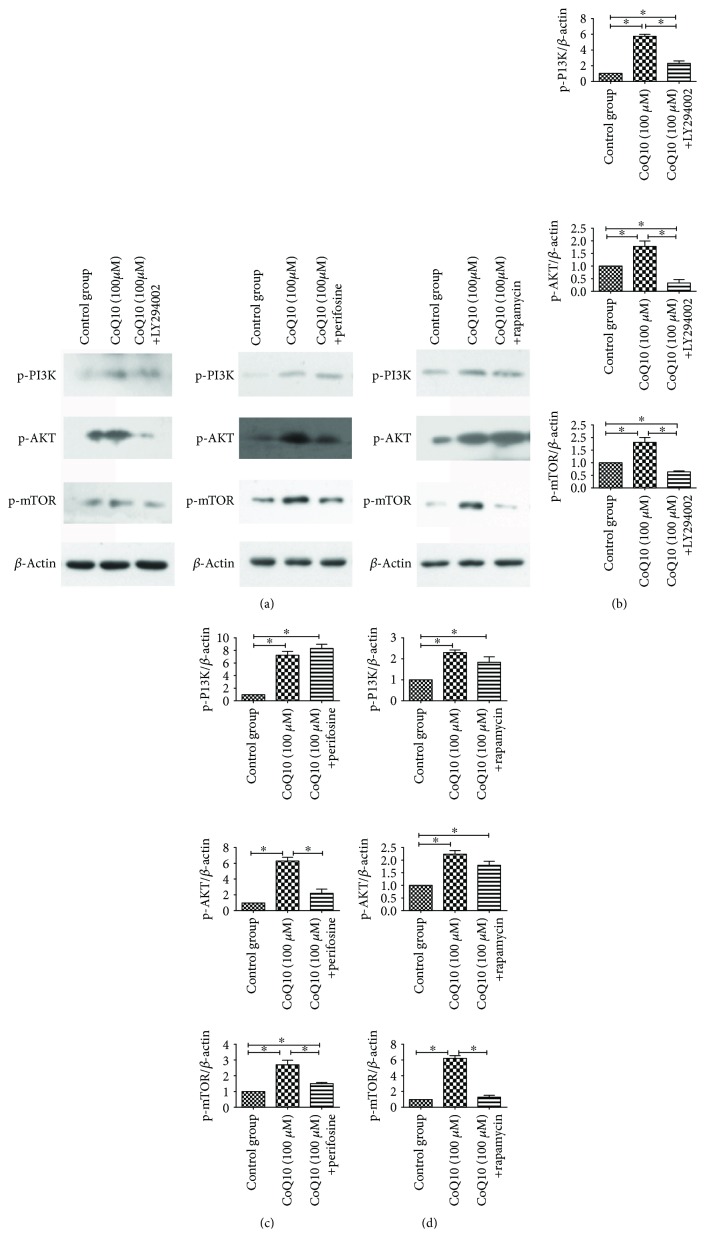
(a) Western blotting analysis for p-PI3K, p-AKT, and p-mTOR expression levels of activated PSCs was performed after 72 h with CoQ10 treatment, as well as the addition of LY294002 (PI3K-specific inhibitor), perifosine (AKT-specific inhibitor), or rapamycin (mTOR-specific inhibitor). (b) Quantification of western blots for p-PI3K, p-AKT, and p-mTOR expression levels in the various groups with the addition of LY294002 compared to the normal control group (^∗^ *P* < 0.05; *N* = 3). (c) Quantification of western blots for p-PI3K, p-AKT, and p-mTOR expression levels in the various groups with the addition of perifosine compared to the normal control group (^∗^ *P* < 0.05; *N* = 3). (d) Quantification of western blots for p-PI3K, p-AKT, and p-mTOR expression levels in the various groups with the addition of rapamycin compared to the normal control group (^∗^ *P* < 0.05; *N* = 3).

**Figure 7:**
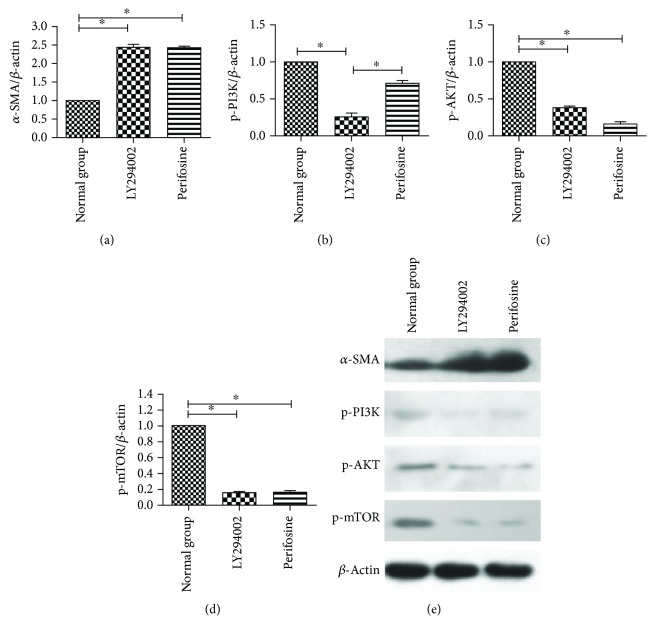
(a) Quantification of western blots for *α*-SMA expression levels in the various groups compared to the normal control group (^∗^ *P* < 0.05; *N* = 3). (b) Quantification of western blots for p-PI3K expression levels in the various groups compared to the normal control group (^∗^ *P* < 0.05; *N* = 3). (c) Quantification of western blots for p-AKT expression levels in the various groups compared to the normal control group (^∗^ *P* < 0.05; *N* = 3). (d) Quantification of western blots for p-mTOR expression levels in the various groups compared to the normal control group (^∗^ *P* < 0.05; *N* = 3). (e) Western blotting analysis for *α*-SMA, p-PI3K, p-AKT, and p-mTOR expression levels with inhibition by Ly294002 and perifosine treatment in PSCs.

Our previous study showed that expression levels of p-PI3K, p-AKT, and p-mTOR were dose-dependently upregulated by CoQ10 in primary PSCs. We suspected that CoQ10 suppressed PSC cell autophagy through the PI3K/AKT/mTOR signaling pathway. Therefore, in this study, we added specific inhibitors of the PI3K-AKT-mTOR signaling pathway to study that underlying mechanism more deeply. After 72 hours of CoQ10 treatment of PSCs, the expression levels of p-PI3K, p-AKT, and p-mTOR increased. With the addition of LY294002, a PI3K-specific inhibitor, the expression levels of p-PI3K, p-AKT, and p-mTOR were significantly lower. After the administration of perifosine, an AKT-specific inhibitor, the expression of p-PI3K was not significantly changed, and the expression levels of p-AKT and p-mTOR were lower. After treatment with rapamycin, an mTOR-specific inhibitor, the expression levels of p-PI3K and p-AKT were not significantly changed, and the expression levels of p-mTOR were lower. These results suggest that CoQ10 suppresses PSC autophagy via the PI3K/AKT/mTOR signaling pathway (Figure 6). The *α*-SMA, p-PI3K, p-AKT, and p-mTOR expression levels with the inhibition by LY294002 and perifosine treatment in PSCs are shown in Figure 7. It indicated that LY294002 and perifosine can increase the expression of *α*-SMA in PSCs. There is no obvious morphological change for PSCs treated with LY294002 and perifosine in this study.

### 3.8. ROS Are Upstream of Autophagy in PSCs

**Figure 8:**
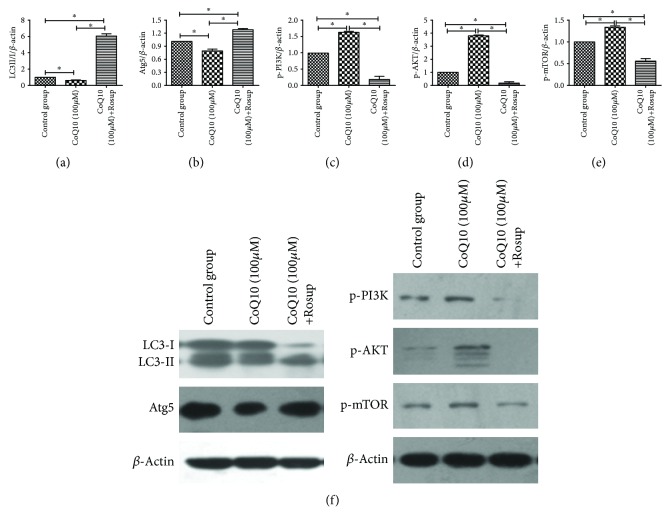
(a) Quantification of western blots for LC3 expression levels in the various groups compared to the normal control group (^∗^ *P* < 0.05; *N* = 3). (b) Quantification of western blots for Atg5 expression levels in the various groups compared to the normal control group (^∗^ *P* < 0.05; *N* = 3). (c) Quantification of western blots for p-PI3K expression levels in the various groups compared to the normal control group (^∗^ *P* < 0.05; *N* = 3). (d) Quantification of western blots for p-AKT expression levels in the various groups compared to the normal control group (^∗^ *P* < 0.05; *N* = 3). (e) Quantification of western blots for p-mTOR expression levels in the various groups compared to the normal control group (^∗^ *P* < 0.05; *N* = 3). (f) Western blotting analysis for LC3, Atg5, p-PI3K, p-AKT, and p-mTOR expression levels of activated PSCs was performed after 72 h with CoQ10 treatment, as well as the addition of Rosup.

With the addition of Rosup, the expression levels of the autophagy biomarkers LC3 and Atg5 were higher. Meanwhile, the levels of p-PI3K, p-AKT, and p-mTOR were lower, suggesting that it is at the level of upstream autophagy that CoQ10 reduces intracellular levels of ROS in PSCs (Figure 8).

## 4. Discussion

Several studies have suggested that the activation of PSCs has an essential role in the progression of pancreatic fibrosis [18, 19]. Any remedies that suppress the activation of PSCs may be potential candidates for therapy strategies in pancreatic fibrosis. The present study verified the beneficial effects of CoQ10 in CP-associated fibrosis. CoQ10 ameliorated pancreatic fibrosis via the ROS-triggered mTOR signaling pathway. All of these results indicated that CoQ10 could be an effective therapy for pancreatic fibrosis.

CP correlates with progressive fibrosis and necrosis [20]. Patients with CP have abdominal pain and endocrine and exocrine insufficiency [21, 22]. Histologically, there are excess collagen deposits and loss of pancreatic islets and acinar cells in CP [23]. Reports on pancreatic fibrosis have shown that PSCs are activated in the course of pancreatic damage, and they are the main sources of collagen in pancreatic fibrosis [24].

Our study demonstrated the suppressive effect of CoQ10 on activated PSCs. CoQ10 significantly inhibited collagen deposition and the production of fibrogenic mediators (*α*-SMA) in the CP animal model. Meanwhile, compared to the respective controls, CoQ10 led to a remarkable amelioration of histological changes, including acinar cell atrophy and vacuolization with complete loss of the cytoplasm and inflammatory cell infiltration.

Following the accumulation of extensive reports on oxidative stress in CP [9], subsequent studies concentrated on antioxidants, and it was shown that patients with CP have poor antioxidant status [25]. It is known that ROS generation precedes downstream cellular cascades, involving autophagy. Depending on the stimulus, autophagy and ROS can regulate one another. There have been many complications in dissecting the interplay between autophagy and ROS in terms of cell fate [26–28].

Our previous study found that CoQ10 reduced intracellular levels of ROS in PSCs [13]. In the present study, in the CP animal model, a significant decrease in tissue ROS production was seen in the pretreatment and posttreatment with CoQ10 groups (*P* < 0.05). Furthermore, there was also a significant increase in MDA levels and decreases in GSH and SOD levels during pretreatment and posttreatment with CoQ10. Because ROS can trigger autophagy, we believe that it is the upstream occurrence of cell autophagy where CoQ10 reduces intracellular levels of ROS in PSCs. Therefore, we performed a rescue experiment using Rosup to test that speculation. We showed that expression levels of autophagy biomarkers were higher and that the levels of PI3K-AKT-mTOR signaling molecules were lower with the addition of Rosup.

In our previous study, we found that CoQ10 correlated with the downregulation of autophagy via the PI3K-AKT-mTOR signaling pathway [13]. These may be the underlying mechanisms for CoQ10 suppressing the activation of PSCs. Therefore, in this study, we performed a rescue experiment using specific inhibitors of the PI3K-AKT-mTOR signaling pathway to investigate that underlying mechanism more deeply.

Currently, CoQ10 is used to treat many diseases. Further studies related to the clinical suppressive effect of CoQ10 for activated PSCs are still needed. To conclude, our study indicated that CoQ10 may be a remedy for clinical therapy of pancreatic fibrosis.

## Data Availability

The data used to support the findings of this study are available from the corresponding author upon request.

## Supplementary Materials

Supplementary Materials Antibodies and conditions used for western blotting analyses and IF staining.
