# Supplementary material for: Coenzyme Q10 Ameliorates Pancreatic Fibrosis via the ROS-Triggered mTOR Signaling Pathway
Source: Oxid Med Cell Longev. 2019 Feb 7;2019:8039694. doi: 10.1155/2019/8039694 (PMC6383547; doi:10.1155/2019/8039694)
Supplement: Supplementary Materials — Antibodies and conditions used for western blotting analyses and IF staining. [file 8039694.f1.docx]

Supplementary Table S1 online

Antibodies and conditions used for western blotting analyses and IF staining.

| Antibody | Number | Species | Dilution | Source |
| --- | --- | --- | --- | --- |
| α-SMA | ab5694 | Rabbit polyclonal antibody | 1:500 | Abcam |
| Atg5 | #12994 | Rabbit monoclonal antibody | 1:1000 | Cell Signaling Technology |
| LC3B | L7543 | Rabbit polyclonal antibody | 1:1000 | Sigma |
| SQSTM1/p62 | #5114 | Rabbit polyclonal antibody | 1:1000 | Cell Signaling Technology |
| β-actin | #4970 | Rabbit monoclonal antibody | 1:1000 | Cell Signaling Technology |
| Phospho-mTOR (Ser2448) | #2971 | Rabbit polyclonal antibody | 1:1000 | Cell Signaling Technology |
| Phospho-Akt (Ser473) | #4060 | Rabbit monoclonal antibody | 1:1000 | Cell Signaling Technology |
| PI3 Kinase p85 (19H8) | #4257 | Rabbit monoclonal antibody | 1:1000 | Cell Signaling Technology |
